# A New Model and Its Application for the Dynamic Response of RGO Resistive Gas Sensor

**DOI:** 10.3390/s19040889

**Published:** 2019-02-20

**Authors:** Hongfei Du, Guangzhong Xie, Yuanjie Su, Huiling Tai, Xiaosong Du, He Yu, Qiuping Zhang

**Affiliations:** 1 School of Optoelectronic Science and Engineering, State Key Laboratory of Electronic Thin Films and Integrated Devices, University of Electronic Science and Technology of China (UESTC), Chengdu 610054, China; hfdu@uestc.edu.cn (H.D.); gzxie@uestc.edu.cn (G.X.); xsdu@uestc.edu.cn (X.D.); yuhe@uestc.edu.cn (H.Y.); zhqpdq@163.com (Q.Z.); 2 School of Mathematical Sciences, University of Electronic Science and Technology of China (UESTC), Chengdu 611731, China

**Keywords:** RGO resistive gas sensor, baseline drift, intermolecular forces based model, FSDE, room temperature

## Abstract

An reduced graphene oxide (RGO) resistive gas sensor was prepared to detect ammonia at room temperature, the result indicated that the desorption of gas (NH${}_{3}$) molecules from a graphene-based sensor was difficult, which lead to a baseline drift. The responses of different concentrations were compared and studied. It was found that both the response rate and its acceleration were affected by the gas concentration. An Intermolecular Forces Based Model was established to explain the adsorption and desorption dynamic response curves. A new method was proposed based on this model. The first and second derivative extrema (FSDE) of the response curve can be attained quickly to calibrate the gas concentrations. The experiment results demonstrated that this new method could eliminate the baseline drift and was capable of increasing the efficiency of gas calibration significantly.

## 1. Introduction

Schedin reported the first graphene based gas sensor in 2007 [1] since the discovery of graphene in 2004 [2]. Graphene has exceptional properties such as large surface area, low electrical noise, good thermal stability, and high carrier mobility at room temperature, etc. [3]. Due to its favorable gas sensing performance, graphene and its derivatives like pristine graphene (PG), graphene oxide (GO), reduced graphene oxide (RGO), etc. have been investigated by many researchers [4,5,6]. GO is an analog of graphene with many functional groups and increased chemical activity, but it is electrically insulating [7,8]. RGO, as a promising material with both high conductance and many chemically active defect sites [7], is inexpensive and easy to prepare and sometimes treated as graphene [6]. Nevertheless, the desorption of gas molecules from graphene based sensor is difficult without UV-light or high operating temperature [1,8,9,10], which leads to a baseline drift (i.e., lack of complete recovery when gas is off) of the sensor at room temperature [7]. There is no suitable model for dynamic response of graphene based gas sensor to solve this problem till now.

The first mathematical fit to an isotherm for gaseous adsorbates was published by Freundlich and Küster (1906). The adsorption isotherm theory of the unimolecular layer was given by Langmuir [11], Brunauer, Emmett, and Teller derived BET theory for multilayer adsorption [12]. G. L. Aranovich and M. D. Donohue developed a statistical mechanical theory of adsorption compression for Lennard–Jones molecules [13].

The dynamic adsorption and desorption process based on Langmuir theory was analyzed by Hu, A. Tételin. [14,15]. Similar models for graphene or RGO were developed by SangZi Liang, Chenyu Wen, and Nowzesh Hasan, etc. [16,17,18]. However, Kisliuk developed the precursor state theory (1957), whereby adsorption of gas molecules to the surface is more likely to occur around gas molecules that are already present on the solid surface [19].

In this paper, RGO was deposited on interdigitated electrodes (IDEs) with airbrush technology to construct resistive sensor for detecting NH${}_{3}$ at room temperature. It was found that the higher gas concentration leaded to a faster response. The response speed is related to the strength of intermolecular attractions and repulsions of adsorbed molecules, so an Intermolecular Forces Based Model of the dynamic response of an RGO resistive gas sensor was established, by using the assumptions of Langmuir theory for reference. The theoretical analyses of the model showed that the FSDE of the dynamic response process could be used to detect the gas concentration rapidly. The experimental data verified this new detection method.

## 2. Experimental

### 2.1. Sensor Preparation

**Figure 1:**
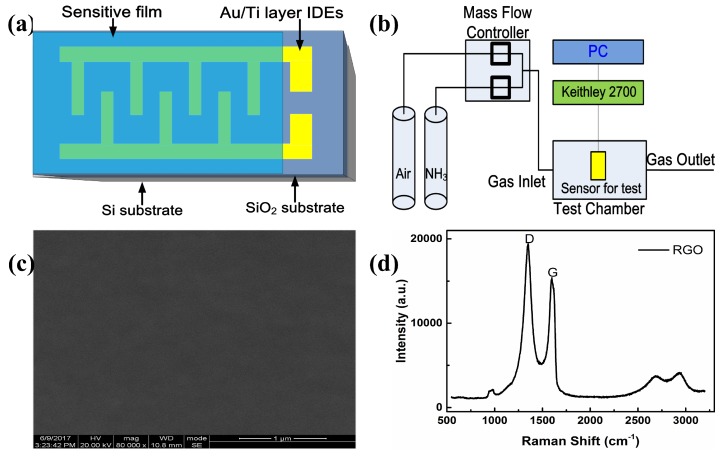
(**a**) Schematic illustration of interdigitated electrodes (IDEs), (**b**) Measurement system, (**c**) scanning electron microscope (SEM) image of reduced graphene oxide (RGO) film, and (**d**) Raman spectra of RGO film.

The RGO aqueous solution (1 mL, 0.43 wt.%, RGO: 96.41% C, 3.59% O, 1–10 layers) was diluted with deionized water (19 mL), and sonicated for 30 min to obtain a uniform dispersion. The RGO solution (0.5 mL) was airbrushed on interdigital electrodes (IDEs), and heated in a vacuum oven at 70 ${}^{\circ }$C for 12 h. Reduced graphene oxide (RGO) aqueous solution was obtained from Chengdu Organic Chemicals Co. Ltd., Chinese Academy of Sciences, Chengdu, China. The IDEs depicted in Figure 1a were fabricated using standard semiconductor technology.

### 2.2. Test Instrument and Measurement Procedure

The measuring system for gas sensing was shown in Figure 1b. Measurement results were obtained at room temperature (300 K). Dry air was used as the carrier, dilution, and purge gas. Gas concentration was controlled by a mass flow controller (MT50-4J, Beijing Metron Instruments Co. Ltd., Beijing, China). The sensor was fixed into a small sealed metal chamber (1.463 cm${}^{3}$) and the total flow rate (air and NH${}_{3}$) was kept at 500 mL/min when the gas (NH${}_{3}$) concentrations were changed (20 ppm raised to 100 ppm, and then lowered to 50 ppm). Electric resistance of the sensor was sampled every 4 s and measured by Keithley 2700 multimeter/Data Acquisition System, and collected real-time by PC with corresponding data acquisition hardware and software. Theoretical study was accomplished with the aid of Mathematica software.

### 2.3. The Characteristics of Film

The scanning electron microscope (SEM) image of RGO thin film was shown in Figure 1c. It could be observed that the surface of RGO film was smooth, indicating the film prepared by airbrush was uniform. Raman spectroscopy was used to characterize the structure of RGO. The Raman spectra in Figure 1d exhibited two prominent peaks at 1348 cm${}^{-1}$ and 1594 cm${}^{-1}$, corresponding to the D and G bands of RGO. The G band was related to the graphitic hexagon-pinch mode, while the D band referred to the structural defects and partially disordered structures in the material [20,21,22].

### 2.4. The Dynamic Response and Preliminary Analysis

In this paper, sensing response *S* was defined as [21]

(1) $$S=R_{0}/R_{t}$$

$R_{0}$ and $R_{t}$ represented respectively the electric resistance when the sensor was exposed to dry air and NH${}_{3}$.

**Figure 2:**
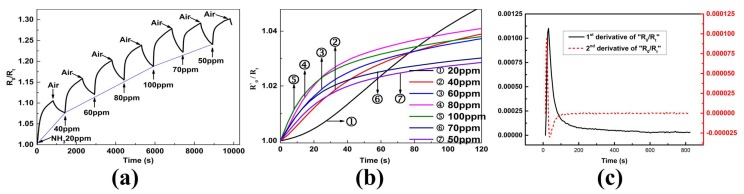
Response analysis. (**a**) Real-time response of the sensor to different NH${}_{3}$ concentrations, (**b**) Combination and comparison of response to different NH${}_{3}$ concentrations, (**c**) First and second derivative of response to 60 ppm NH${}_{3}$.

Figure 2a displayed the real-time responses of RGO sensor exposure to NH${}_{3}$. The response increased as NH${}_{3}$ was induced, and decreased as NH${}_{3}$ was ceased. The response curve was smooth, indicating that the sensor made of RGO had less signal noise. The desorption of NH${}_{3}$ molecules from RGO film was difficult resulting in a baseline drift. The baseline drift makes the response increasing whether the gas concentration rose or fell down. The maxima of the response were used to characterize the gas concentrations according to conventional method, which is not applicable here due to baseline drift.

A phenomenon was also found here: the higher the ammonia concentration, the faster the response changed. This could be seen more easily from Figure 2b, which calculated the response of each adsorption process separately by defining the initial resistance of each adsorption process as ${R^{\prime }}_{0}$. In other words, the value of $R_{0}/R_{t}$ was subtracted at the introduction of NH${}_{3}$ during each step change. It was speculated that both the rate and the acceleration of response were affected by the gas concentrations regardless the baseline drift.

## 3. Theory

### 3.1. Influence of Adsorbed Gas Molecules

As NH${}_{3}$ gas molecules are adsorbed on the RGO film, electrons are released into the conduction band and resistance $R_{t}$ is changed [2,3]. The carrier concentration *n* is affected by the number *N* of adsorbed NH${}_{3}$ molecules, n∝N. $R_{t}$ is proportional to the resistivity ρ, and the inverse of ρ is electrical conductivity σ=1/ρ=neμ, where *e* is one electron charge, μ is mobility of charge carriers. So the change of resistance is influenced by the number of adsorbed gas molecules

$$R_{t}\propto \rho \propto 1/n\propto 1/N$$

According to Equation (1), sensing response *S* is proportional to adsorbed gas molecules’ number *N*

(2) S∝N

The adsorption process is generally classified as physisorption and chemisorption, the former is related to the intermolecular force and the latter is characterized by covalent bonding [23]. Although the graphene sensors often function via chemisorption of gas molecules, chemisorption takes place after physisorption, so we can focus on physisorption (characteristic of intermolecular force). Figure 2c displayed the first and second derivative curves of response on exposure to 60 ppm NH${}_{3}$, there was one extremum in the first derivative curve and two extrema in the second derivative curve. The major changes are concentrated in a short period of time, so we can assume that physisorption is the main factor at this stage. The factors affecting the adsorption are mainly the adsorbate-adsorbent attraction (i.e., the attraction between RGO and NH${}_{3}$ molecules) at the initial state of gas injection, and then mainly the intermolecular force. Since the intermolecular force is mainly Van der Waals force, when there are few molecules on the surface of the RGO film, the interaction between these gas molecules is mainly attractive force, causing more molecules to be absorbed on the surface of the film. When there are many molecules on the film, the intermolecular forces are mainly repulsive force, resulting in reduced adsorption rate that finally attained a certain balance.

### 3.2. Basic Assumptions

In order to simplify the problem, this article draws on the Langmuir gas-solid adsorption theory [14,15] and Kisliuk’s theory [19], which gives the following assumptions:

1. Surface approximation. The surface of the film is homogeneous and the adsorption process is deemed as monolayer adsorption. This assumption implies that multilayer and microporous adsorption, etc., are not considered.
2. Gas concentration effect. The adsorption and desorption of gas molecules occur simultaneously, and their numbers are influenced by the number of molecules contacting the surface of the film per unit time. This assumption means that the main factor that affects the adsorption and desorption is the gas concentration under the conditions of constant temperature and pressure, with no illumination.
3. Response process approximation. When gas concentration is changed, the number of molecules colliding on the surface of the film changes greatly at the beginning, thereafter the adsorption and desorption occur simultaneously and at last reach a balance state. The main factor that affects the adsorption and desorption is the gas intermolecular force.

The kinetics of adsorption and desorption can be given as the following model.

### 3.3. Intermolecular Forces Based Model of Adsorption and Desorption

The following discussion considers only the gas to be measured (for example, NH${}_{3}$). The total number of gas molecules per cm${}^{3}$ in the detection chamber, $N_{G}$, can be regarded as a constant when absolute temperature, pressure, and gas concentration *c* do not change. Furthermore, $N_{G}$ is proportional to *c*, which means $N_{G}\propto c$.

**Figure 3:**
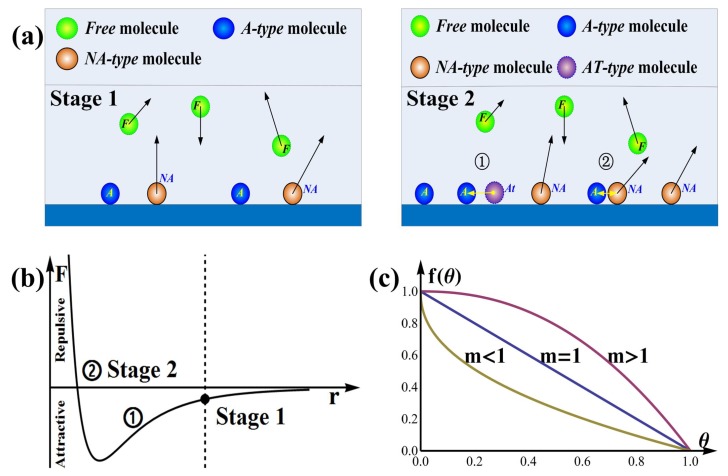
(**a**) Schematic diagrams of adsorption, Stage 1: $N_{0}$ gas molecules (*A-type*) are adsorbed on the surface; Stage 2: the influence of intermolecular forces, attractive at long distances and repulsive at short distances, (**b**) The relationship between intermolecular forces (*F*) and distance (*r*), (**c**) The variety trend of f(θ).

As shown in Figure 3a stage 1, when the gas is induced, a part of the gas molecules hit on the film surface, whose number is $N_{S}$, leaving the rest molecules as *Free molecules*. When the external conditions do not change, $N_{S}$ is a constant, and is proportional to gas concentration *c*, $N_{S}\propto c$.

The gas molecules ($N_{S}$) which hit on the surface of sensor are adsorbed partly, leaving the others reflecting back. The Adsorbed-molecules are also called *A-type molecules*; the others are called Not-Adsorbed-molecules or *NA-type molecules*. So

Total molecules $N_{G}$= *Free* molecules + molecules hit on the film surface ($N_{S}$)= *Free* molecules + *A-type* molecules + *NA-type* molecules

The sensor’s response is affected by the number of *A-type* molecules N(t).

The adsorption and desorption process can be discussed by considering the effect of intermolecular forces, where attractive force dominates at long distances while repulsive force dominates at short distances, as shown in Figure 3b. When a *NA-type* molecule is close to an *A-type* molecule, as shown in Figure 3a stage 2 ①, it is attracted to the film by attractive force, this is called attracted molecule or *AT-type molecule*, and becomes *A-type* molecule at last. If the distance of two molecules on the film is too short, one of them will be repelled away from the film surface by repulsive force and become *NA-type molecule* as shown in Figure 3a stage 2 ②.

Let $\theta \left(t\right)=N\left(t\right)/N_{\infty }$, θ(t) is the surface coverage, N(t) is *A-type* molecules’ number, and $N_{\infty }=N\left(\infty \right)$ is the number when balance is reached.

Then, the adsorption process can be divided to the following two stages:

1. Stage 1: The Beginning State
  This stage can finish instantaneously under ideal conditions.
  The randomly moving gas molecules collide constantly with each other and with the surface of sensor, $N_{S}$ of them hitting on the surface of sensor, and $\alpha \cdot N_{S}$ molecules are adsorbed on the surface. The adsorption ratio α is mainly affected by the sensitive material, surface morphology, temperature, humidity, pressure, illumination, and gas species, etc. This state is shown in Figure 3a stage 1 and Figure 3b stage 1. $N_{0}=N\left(0\right),\theta_{0}=\theta \left(0\right)=N_{0}/N_{\infty }$.
2. Stage 2: Adsorption Process
  Adsorption and desorption occur simultaneously, however, we can focus only on the combined effect, which can be regarded as net increment of adsorption.
  (1) With the increment of *A-type* molecules, the probability that *NA-type* molecules meet *A-type* molecules increases. Meanwhile, the average intermolecular distance decreases and the attractive force increases as shown in Figure 3b stage 2 ①. Consequently, some of the *NA-type* molecules approaching *A-type* molecules are more likely to be attracted to the film and transferred to *AT-type* molecules as shown in Figure 3a stage 2 ①. Note that the number of the *NA-type* molecules is proportional to molecules hitting on the film surface ($N_{S}$), then the rate of adsorption is affected by $N_{S}$ and a function g(θ)
  $$\frac{d\theta }{dt}\propto N_{S}\cdot g\left(\theta \right)$$
  where g(θ) is an increasing function of θ.
  (2) With the increment of the *A-type* molecules number, the average distance between some *A-type* molecules decreases, resulting in the weakening of the attractive forces and the strengthening of the repulsive forces as shown in Figure 3b stage 2 ②. As a result, the number of *AT-type* molecules will decrease, which means the adsorption rate will slow down
  $$\frac{d\theta }{dt}\propto f\left(\theta \right)$$
  where f(θ) is a decreasing function of θ.

The gas molecules move randomly, some of them are attracted to the film and some of them are repelled away from the film, so the circumstances of Figure 3a stage 2 ① ② and Figure 3b stage 2 ① ② coexist throughout the whole adsorption process. Thus, the elementary model can be obtained

$$\frac{d\theta (t)}{dt}=k_{0}\cdot N_{S}\cdot f\left(\theta \left(t\right)\right)\cdot g\left(\theta \left(t\right)\right)$$

Here $k_{0}$ is a coefficient.

The intermolecular force does not vary linearly with the average intermolecular distance, therefore f(θ) may have the shape as displayed in Figure 3c, which can be approximated as

$$f\left(\theta \right)=1-\theta^{m}$$

Here *m* is a constant that is affected by intrinsic mechanisms and extrinsic factors (environment), the former is mainly determined by devices and gas sensitive material and the latter is mainly included absolute temperature *T*, light, and pressure *P* etc.

In the simplest case of adsorption, g(θ) is the fractional coverage θ [14], then the kinetics of the adsorption are described by

(3) $$\frac{d\theta }{dt}=k_{a}\cdot \theta \cdot \left(1-\theta^{m}\right)$$

Here $k_{a}=k_{0}\cdot N_{S}\propto c$.

The more general expression is $d\theta /dt=k_{A}\theta^{n}-k_{D}\theta^{m}$, $k_{A},k_{D}$ and *n* are constants. However, the figure of $k_{A}\theta^{n}-k_{D}\theta^{m}$ has no essential difference with that of $k_{a}\theta \left(1-\theta^{m}\right)$, and $d\theta /dt=k_{a}\theta \left(1-\theta^{m}\right)$ is a Bernoulli differential equation that can be solved explicitly. So Equation (3) is selected as our differential model. Since $\theta \left(0\right)=\theta_{0}$, Equation (3) can be solved analytically

$$\theta^{-m}=1+\left(\theta_{0}^{-m}-1\right)e^{-k_{a}mt}$$

Let $b=\theta_{0}^{-m}-1$, the general solution is Equation (4)

(4) $$\theta \left(t\right)={\left(1+be^{-k_{a}mt}\right)}^{-\frac{1}{m}}$$

The desorption process can be obtained similarly. When the target gas is released, there will be a significant reduction of the adsorbed molecules because the impacted molecules reduce greatly. The rate of desorption will increase as the repulsive force plays a dominant role at the initial stage. However, intermolecular attraction will gradually dominate the process, and desorption will slow down with the decrease of the molecules number on the surface. Let *v* be a parameter similar to *m*, the desorption process can be expressed as

$$\frac{d\theta }{dt}=-k_{d}\cdot \theta \cdot \left(1-\theta^{v}\right)$$

(5) $$\theta \left(t\right)={\left(1+be^{k_{d}vt}\right)}^{-\frac{1}{v}}$$

Here, $k_{d}$ is a coefficient of desorption. The main difference between the desorption model Equation (5) and adsorption model Equation (4) is that the coefficient of the desorption process.

### 3.4. Analysis of Intermolecular Forces Adsorption Model

For adsorption process, the first derivative of Equation (4) is

(6) $$\theta^{\prime }\left(t\right)=\frac{d\theta }{dt}=bk_{a}\frac{{\left(1+be^{-k_{a}mt}\right)}^{-1/m}}{b+e^{k_{a}mt}}$$

The second derivative of Equation (4) is

(7) $$\theta^{\prime \prime }\left(t\right)=\frac{d^{2}\theta }{dt^{2}}=bk_{a}^{2}\frac{{\left(1+be^{-k_{a}mt}\right)}^{-1/m}}{{\left(b+e^{k_{a}mt}\right)}^{2}}\left(b-me^{k_{a}mt}\right)$$

The third derivative of Equation (4) is

(8) $$\theta^{\prime \prime \prime }\left(t\right)=\frac{d^{3}\theta }{dt^{3}}=bk_{a}^{3}\frac{{\left(1+be^{-k_{a}mt}\right)}^{-1/m}}{{\left(b+e^{k_{a}mt}\right)}^{3}}\left(b^{2}-bm\left(3+m\right)e^{k_{a}mt}+m^{2}e^{2k_{a}mt}\right)$$

The first derivative has one extremum and the second derivative has two extrema. The first and second derivative extrema (FSDE) are analyzed as the following.

(1) The first derivative’s extremum can be obtained if the second derivative equal to 0. Let $t_{1}$ be the time to reach the extremum, the result can be obtained according to Equation (7).

$$\theta^{\prime \prime }\left(t\right)=0⟹b-me^{k_{a}mt}=0⟹t_{1}=\frac{1}{k_{a}m}\ln\left(\frac{b}{m}\right)$$

When $t_{1}=1/\left(k_{a}m\right)\ln\left(b/m\right)$, the first derivative’s extremum of Equation (6) is

$$\theta_{max}^{\prime }=k_{a}m{\left(1+m\right)}^{-\frac{1+m}{m}}$$

Since $k_{a}\propto c$, *m* is constant and $b=\theta_{0}^{-m}-1$, $\theta_{max}^{\prime }\propto c$. According to Equation (2), $S\propto N=N_{\infty }\cdot \theta$, the following conclusions can be attained

(9) $$S_{max}^{\prime }\propto c$$

That means the extremum’s value of the first derivative is proportional to the gas concentration *c*; the initial coverage $\theta_{0}$ influences only the time to achieve the extremum $t_{1}$.

(2) The second derivative’s extrema can be obtained if the third derivative (Equation (8)) is equal to 0. The maximum and minimum of the second derivative of Equation (7) can be obtained similarly

$$\theta_{max}^{\prime \prime }=k_{a}^{2}\frac{2m^{2}\left(-1-m+\sqrt{5+6m+m^{2}}\right){\left(1+\frac{2m}{3+m-\sqrt{5+6m+m^{2}}}\right)}^{-\frac{1}{m}}}{{\left(-3-3m+\sqrt{5+6m+m^{2}}\right)}^{2}}$$

$$\theta_{min}^{\prime \prime }=k_{a}^{2}\frac{2m^{2}\left(-1-m-\sqrt{5+6m+m^{2}}\right){\left(1+\frac{2m}{3+m-\sqrt{5+6m+m^{2}}}\right)}^{-\frac{1}{m}}}{{\left(3+3m+\sqrt{5+6m+m^{2}}\right)}^{2}}$$

Since k∝c and *m* is constant, $\sqrt{\theta_{max}^{\prime \prime }}\propto c,\sqrt{\left|\theta_{min}^{\prime \prime }\right|}\propto c$, we can draw the following conclusion

(10) $$\sqrt{S_{max}^{\prime \prime }}\propto c,\sqrt{\left|S_{min}^{\prime \prime }\right|}\propto c$$

That means the square root of the maximum or the absolute value of minimum of the second derivative is proportional to the gas concentration *c*, and not affected by the initial coverage $\theta_{0}$.

## 4. Results and Discussion

### 4.1. New Calibration Method of Gas Concentration

**Figure 4:**
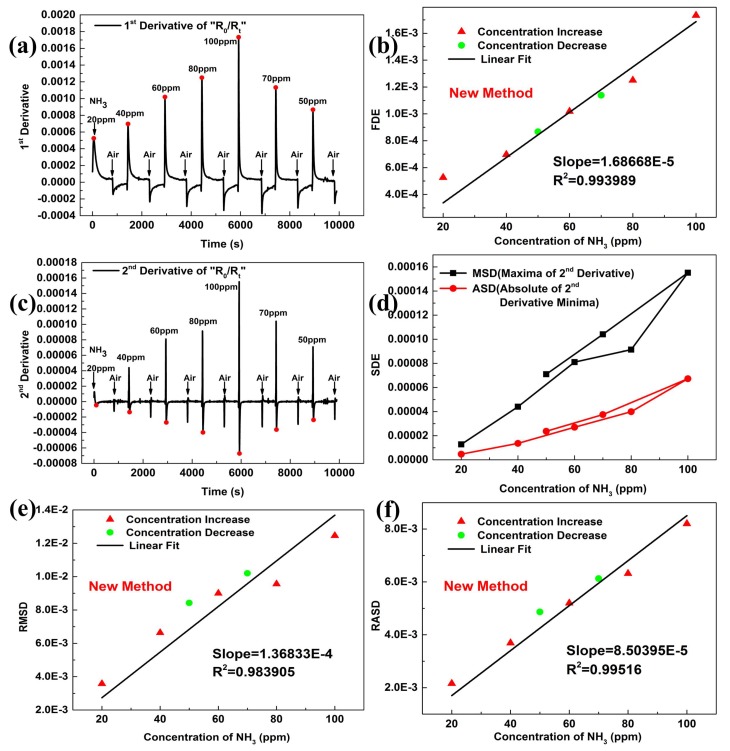
New method. (**a**) First derivative of kinetic response, (**b**) Linear fit of FDE (the First Derivative Extrema) to different NH${}_{3}$ concentrations, (**c**) Second derivative of real-time response, (**d**) SDE (Second Derivative Extrema) to different NH${}_{3}$ concentrations, (**e**) Linear fit of RMSD (the square Root of Maxima of Second Derivative) to different NH${}_{3}$ concentrations, (**f**) Linear fit of RASD (the square Root of Absolute minima of Second Derivative) to different NH${}_{3}$ concentrations.

The first derivative of dynamic response was shown in Figure 4a. The first derivative extrema (FDE) showed a good linear relationship with the gas concentrations, as exhibited in Figure 4b. The linear fit verified $S_{max}^{\prime }\propto c$. Similar cases can also be found in other references, where the peak values of the first derivative were suggested to measure gas concentrations before getting saturated [24,25,26].

The above analysis showed that the rate of response was affected by the gas concentrations, so the acceleration of response was also analyzed. Figure 4c indicated that the second derivative of dynamic response had peak and valley values, and both of them were highly correlated with gas concentrations, as shown in Figure 4d. The FSDE were useful and selected as the features for E-nose [27,28].

The square Root of Absolute minima of Second Derivative (RASD) and square Root of Maxima of Second Derivative (RMSD) exhibited a good linearity with the gas concentrations, as exhibited in Figure 4e,f, and this verified $\sqrt{S_{max}^{\prime \prime }}\propto c,\sqrt{\left|S_{min}^{\prime \prime }\right|}\propto c$.

Being confirmed by the experimental results, the model provided theoretical support for the new gas calibration method, which can be used to calibrate gas concentration.

The new method was not affected by the baseline drift as Figure 4a,c showed. The $R^{2}$ of fitting lines as shown in Figure 4b,e,f indicated that the new method would be idea to evaluate the response.

Moreover, the maxima of the second derivative appeared about 9 s after the gas was imported, the maximum of the first derivative appeared about 18 s, and the minima of the second derivative appeared about 27 s. Using the FSDE to characterize the gas concentrations can increase the calibration speed of the RGO gas sensor.

### 4.2. Influence of Parameter m

**Figure 5:**
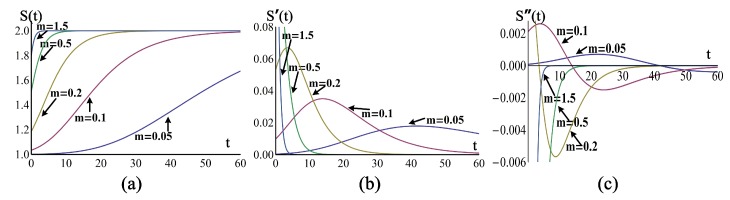
The influence of different parameter *m*. (**a**) The kinetic adsorption response S(t), (**b**) First derivative $S^{\prime }\left(t\right)$, and (**c**) Second derivative $S^{\prime \prime }\left(t\right)$.

*m* is an essential parameter in our model. To reveal the influence of *m*, a simplified simulation is given as follow:

Let S(t)=1+θ(t), where θ(t) is given in Equation (4), b=0.4,ka=1, and *m* varies from 0.05 to 1.5, the shapes of S(t) were shown in Figure 5a. Their first derivative $S^{\prime }\left(t\right)$ and second derivative $S^{\prime \prime }\left(t\right)$ were shown in Figure 5b,c respectively.

When *m* became larger, the response S(t) would become quicker as shown in Figure 5a, and could be deemed as an exponential function if *m* was large enough, for example m=1.5. Furthermore, the first and second derivatives were both like exponential functions when *m* was large enough.

As a consequence, the Intermolecular Forces Based Model may be suitable for more situations of kinetic gas adsorption process.

## 5. Conclusions

The response of an RGO resistance gas sensor was theoretically and experimentally investigated under different ammonia concentrations in this paper. The results revealed that the response speed and acceleration of the sensor were obviously related to the concentration of ammonia.

Then an adsorption model of gas molecules based on the Intermolecular Force was established to analyze the dynamic response of the RGO resistance gas sensor. A new method was proposed based on this model. The FSDE of dynamic response were used to calibrate gas concentrations. The experiment results demonstrated that this new method was propitious to eliminate the baseline drift and the fitting lines had good linearity. The characteristic values can be attained quickly before attaining the adsorption balance. This means the gas concentration can be judged in an extremely short period of time instead of the complete reaction cycle.
